# The patterning of retinal horizontal cells: normalizing the regularity index enhances the detection of genomic linkage

**DOI:** 10.3389/fnana.2014.00113

**Published:** 2014-10-21

**Authors:** Patrick W. Keeley, Benjamin E. Reese

**Affiliations:** ^1^Neuroscience Research Institute, University of California at Santa BarbaraSanta Barbara, CA, USA; ^2^Department of Molecular, Cellular and Developmental Biology, University of California at Santa BarbaraSanta Barbara, CA, USA; ^3^Department of Psychological and Brain Sciences, University of California at Santa BarbaraSanta Barbara, CA, USA

**Keywords:** nearest neighbor, Voronoi domain, packing factor, retinal mosaic, QTL, recombinant inbred strain, haplotype, principal component analysis

## Abstract

Retinal neurons are often arranged as non-random distributions called “mosaics,” as their somata minimize proximity to neighboring cells of the same type. The horizontal cells serve as an example of such a mosaic, but little is known about the developmental mechanisms that underlie their patterning. To identify genes involved in this process, we have used three different spatial statistics to assess the patterning of the horizontal cell mosaic across a panel of genetically distinct recombinant inbred strains. To avoid the confounding effect of cell density, which varies twofold across these different strains, we computed the “real/random regularity ratio,” expressing the regularity of a mosaic relative to a randomly distributed simulation of similarly sized cells. To test whether this latter statistic better reflects the variation in biological processes that contribute to horizontal cell spacing, we subsequently compared the genomic linkage for each of these two traits, the regularity index, and the real/random regularity ratio, each computed from the distribution of nearest neighbor (NN) distances and from the Voronoi domain (VD) areas. Finally, we compared each of these analyses with another index of patterning, the packing factor. Variation in the regularity indexes, as well as their real/random regularity ratios, and the packing factor, mapped quantitative trait loci to the distal ends of Chromosomes 1 and 14. For the NN and VD analyses, we found that the degree of linkage was greater when using the real/random regularity ratio rather than the respective regularity index. Using informatic resources, we narrowed the list of prospective genes positioned at these two intervals to a small collection of six genes that warrant further investigation to determine their potential role in shaping the patterning of the horizontal cell mosaic.

## INTRODUCTION

The organizing principles by which neurons of a given type are distributed within a structure in the central nervous system have gone largely unexplored. The retina is the primary exception to this, where neuronal populations have been shown to be arranged in non-random distributions known as “mosaics” (Wässle and Riemann, 1978). The patterning present in these mosaics arises from local interactions between neighboring cells of the same type that prohibit close proximity, and can be simulated using minimal distance spacing rules constraining random distributions of cells (Eglen, 2006). While the biological processes that underlie these spacing rules have been elucidated, and may vary depending upon the type of neuron (Reese and Keeley, 2014), the molecular mechanisms responsible for their execution have only recently been addressed (Kay et al., 2012).

Several spatial statistics have been employed to study the orderliness of such retinal mosaics, including the analysis of nearest neighbor (NN) distances and Voronoi domain (VD) areas (Cook, 1996; Galli-Resta et al., 1997). The frequency distribution of these measures for many orderly retinal mosaics approximates a Gaussian distribution, whereas those derived from random simulations of cells have a more Poisson distribution. One commonly used shorthand for describing the “regularity” in such orderly distributions has been to determine the mean NN distance or VD area within a sampled field and divide it by the SD (Wässle and Riemann, 1978; Raven and Reese, 2002). Commonly described as the “regularity index,” such computed ratios will be larger for Gaussian distributions relative to density-matched random distributions. In this manner, real retinal mosaics have been shown to be more regular than random distributions, and the magnitude of the regularity index is assumed to have some biological relevance for the orderliness in such mosaics.

We have argued elsewhere that the degree of regularity in a retinal mosaic should be assessed relative to a density-matched random distribution of similarly sized cells, rather than to a random distribution of points (Reese and Keeley, 2014), because the physical size of the cells constrains spatial positioning. As either density or soma size increases, so does the degree of regularity achieved by a random distribution of cells, and it is the difference from such a random simulation that should be critical for understanding the processes contributing to the formation of regularity. In the present study, we have explored this relationship explicitly, analyzing the regularity indexes of the mosaic of horizontal cells in the mouse retina across three strains of mice, the C57BL/6J strain, the A/J strain, and their F1 cross, each of which varies in the density of these cells. We show that by normalizing each regularity index relative to density-matched random distributions constrained by soma size, achieved by computing a “real/random regularity ratio,” we enhance the differences between the strains that should more acutely reflect the biological processes contributing to this patterning.

A recent study demonstrated that variation in the NN regularity index for the mosaic of cholinergic amacrine cells across a panel of 25 genetically distinct recombinant inbred mouse strains can be mapped to a discrete genomic locus. This suggests that the regularity index of a mosaic reflects a biological process or processes at work. Indeed, that study identified a candidate genetic contributor that, when rendered non-functional, reduced the mosaic regularity of the neuronal population (Keeley et al., 2014b). In the present study, we examined the NN regularity index and VD regularity index of the population of horizontal cells across this same panel of recombinant inbred strains. We then sought validation of the above normalization procedure for the regularity index, asking whether the real/random regularity ratio showed heightened linkage to the variation in genotype across the strains. Finally, we compared the results to another measure of spatial patterning, the “packing factor” (Rodieck, 1991). Using all three measures, we demonstrate that the variation in the patterning of horizontal cells is associated with two genomic loci on Chromosomes (Chrs) 1 and 14.

## MATERIALS AND METHODS

Adult retinas, between 1 and 3 months of age, were examined from the following strains: the C57BL/6J (B6/J hereafter) and A/J parental strains, the B6AF1 cross, and 25 strains of the AXB/BXA recombinant inbred strain-set. The data for these strains were derived from digitized images from a previous study examining the variation in horizontal cell number across these strains; details of the tissue harvesting, immunofluorescence, and microscopy are provided therein (Whitney et al., 2011). All retinal tissues harvested from mice were collected in accord with AVMA guidelines and under authorization by the Institutional Animal Use and Care committee at the University of California, Santa Barbara.

Each retina was sampled at four central and four peripheral locations surrounding the optic nerve head (i.e., two fields in each retinal quadrant). The sampled fields were 225,802 sq. μm in area, with an aspect ratio of 1:1.25, and had a total number of horizontal cells ranging from 93 to 413, depending upon the strain (mean = 205). The X,Y coordinates of every calbindin-positive horizontal cell were determined, from which we computed the NN distance and VD area for every cell in each field, excluding those cells along the border with uncertain NN distances or VD areas. The regularity index for each statistic was calculated for each field by dividing the mean NN distance or VD area by the SD. The eight regularity indexes for a given retina were then averaged to produce the average regularity index for a given animal (sampling only one retina per mouse), with multiple animals being sampled for each strain. The number of mice sampled in each strain is indicated in the histograms. For each real field, a random field, being 225,625 sq. μm in area (475 μm × 475 μm), matched in density and constrained by average soma diameter (9.1 ± 0.7 μm; mean ± SD), was generated and similarly analyzed, to permit a direct comparison to the regularity index that would be achieved from a random distribution of horizontal cells of the same density. The real/random regularity ratio was computed by dividing the regularity index for a given mouse by the average regularity index of its density-matched random simulations. Additionally, for each sampled field, the packing factor was calculated. A value on a bounded scale between 0 and 1, the packing factor describes the extent to which a mosaic approximates a hexagonal lattice, with a value of 0 representing a random mosaic of dimensionless points and a value of 1 representing a perfect lattice. The packing factor was calculated by dividing the effective radius derived from the density recovery profile by the theoretical maximum radius that could be achieved by a lattice of the same density (Rodieck, 1991).

The variation in the regularity indexes, the real/random regularity ratios, and the packing factor across the recombinant inbred and parental strains was mapped to the variation in strain haplotype across the genome using the simple interval mapping tool of GeneNetwork^1^, yielding a likelihood ratio statistic (LRS) for assessing linkage between phenotype and genotype. GeneNetwork computes 2000 permutations of the strain data to compute suggestive (*p* < 0.63) and significant (*p* < 0.05) thresholds for the LRS as another means of assessing the relative probabilities that any quantitative trait locus (QTL) contains a causal gene contributing to the variation in each trait. GeneNetwork also computes 2000 bootstrap tests to determine the relative robustness of each QTL detected. Principal component analysis (PCA) was also performed in GeneNetwork to determine the eigenvector that best accounts for the variance across the NN real/random regularity ratio, VD real/random regularity ratio, and packing factor; before performing the PCA, GeneNetwork normalized the data such that the distribution of each trait had a mean of zero and a SD of one. The first principal component derived from this analysis was then used as a novel quantitative trait that was subsequently mapped. All datasets were deposited in the AXB/BXA phenotypes database of GeneNetwork under accession ID #10282 (horizontal cells, nearest neighbor regularity index), #10283 (horizontal cells, Voronoi domain regularity index), #10288 (horizontal cells, nearest neighbor real/random regularity ratio), #10289 (horizontal cells, Voronoi domain real/random regularity ratio), #10291 (horizontal cells, packing factor), and #10292 (horizontal cells, patterning PCA). All positional data are relative to the NCBI37/mm9 build of the mouse genome.

## RESULTS

**FIGURE 1:**
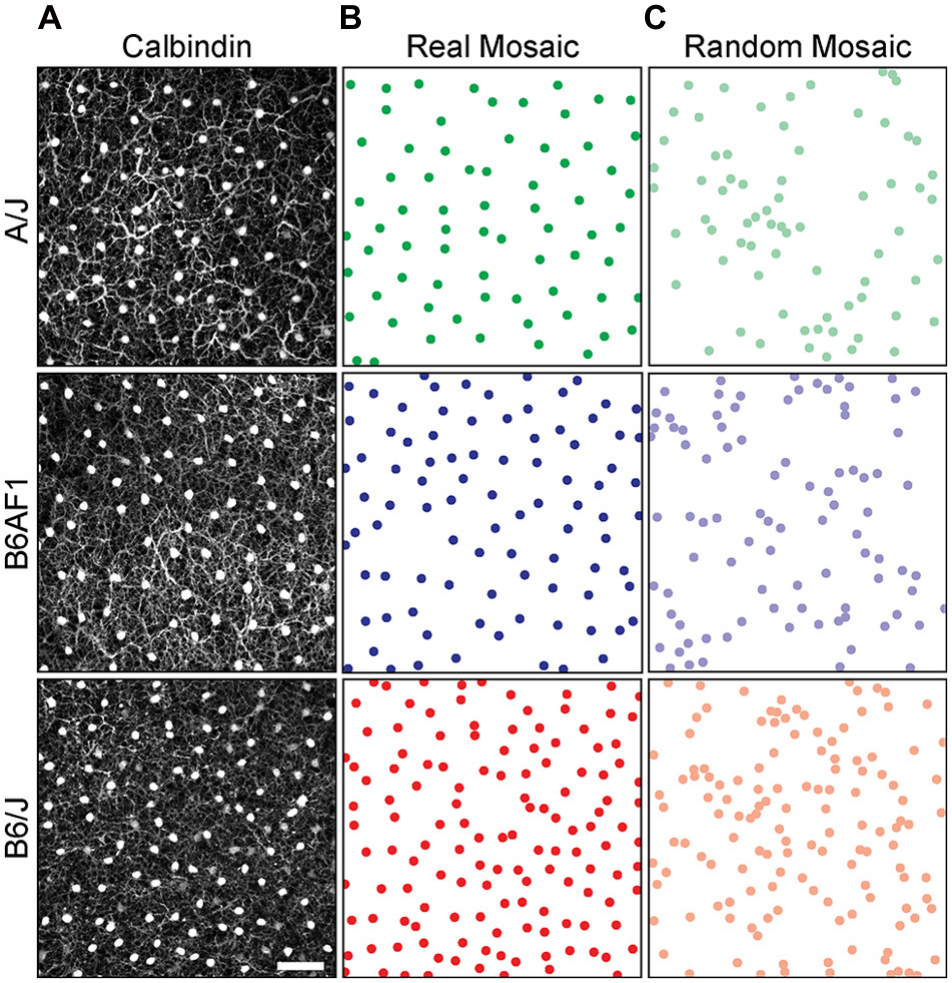
**(A)** Sample fields from the A/J (green), B6AF1 (blue), and B6/J (red) strains, immunolabeled with an antibody to calbindin to reveal the retinal horizontal cells. **(B,C)** Their somal distributions, alongside density-matched random distributions constrained by soma size, are shown for direct comparison. Calibration bar = 100 μm.

**FIGURE 2:**
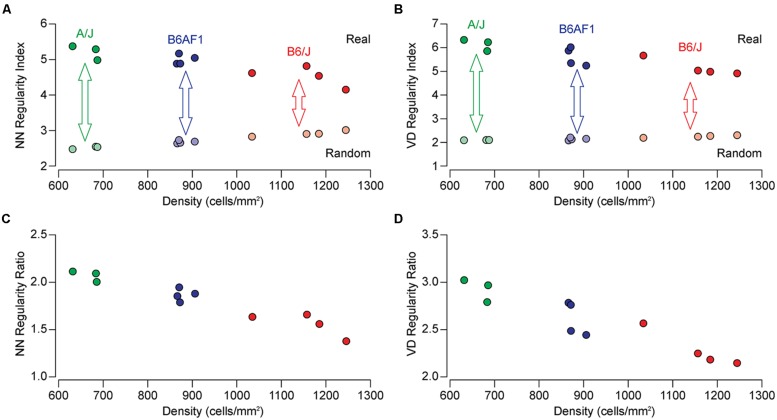
**(A,B)** The NN and VD regularity indexes for individual mice of the A/J (green), B6AF1 (blue), and B6/J (red) strains as a function of cell density (indicated by the saturated colored symbols across the top). Each regularity index is the average of eight sampled fields per retina, while the density indicated is the average density for those eight fields. The regularity index for random simulations of the same average density (and constrained by soma size) are shown for comparison (indicated by the de-saturated colored symbols across the bottom). Notice the increase in regularity for the random distributions as a function of increasing density. **(C,D)** The NN and VD real/random regularity ratio (being the real regularity index divided by the random regularity index) for each retina presented in **(A,B)**. A ratio of 1.0 would signify a mosaic that is no different from a random distribution matched in density and constrained by soma size. By normalizing the regularity index in this manner, the differences between the strains are enhanced.

The population of horizontal cells exhibits substantial variation across different mouse strains, showing a nearly twofold variation in number. The B6/J strain contains nearly twice as many horizontal cells as does the A/J strain, while their F1 cross (B6AF1) falls almost equally between them (Raven et al., 2005a). **Figure 1** illustrates sample fields taken from each of these three strains, along with density- and size-matched random simulations for direct comparison. It is immediately apparent that the patterning in the real mosaics is distinct from those random simulations, their cells being more regularly distributed. Less obvious from the sample fields is any difference in their regularity, but if we compute the NN regularity index for multiple retinas from each strain, they appear to differ, with the lowest density A/J strain being the most regular, having an average NN regularity index of 5.30, followed by the B6AF1 strain, having a slightly lower regularity index of 5.00, while the B6/J strain, containing the greatest density of horizontal cells, having the lowest regularity index of 4.56 (**Figure 2A**). A similar difference was seen for the VD regularity index, as the A/J, B6AF1, and B6/J strains achieved average regularity indexes of 6.14, 5.62, and 5.15, respectively (**Figure 2B**).

The regularity indexes for density-matched, soma-size constrained, random simulations are also shown, for each of the same mice, in **Figures 2A,B** as de-saturated colored symbols. These random simulations have regularity indexes extending from ∼2.0 to 3.0, being more regular than theoretical random distributions associated with dimensionless points (Cook, 1996). Note though that these random distributions differ between the strains, climbing as a function of increasing cell density, due to the space-occupying nature of the cells (although to a lesser extent for the VD analysis). As a consequence, the differences in the patterning between the strains, relative to what they would achieve were they randomly distributed, must be under-appreciated when comparing their regularity indexes alone, particularly for the NN analysis. If, however, we normalize each regularity index by taking into consideration this density-dependency of the random simulations (dividing the former by the latter to compute the real/random regularity ratio), the strain differences become more conspicuous (**Figures 2C,D**).

**FIGURE 3:**
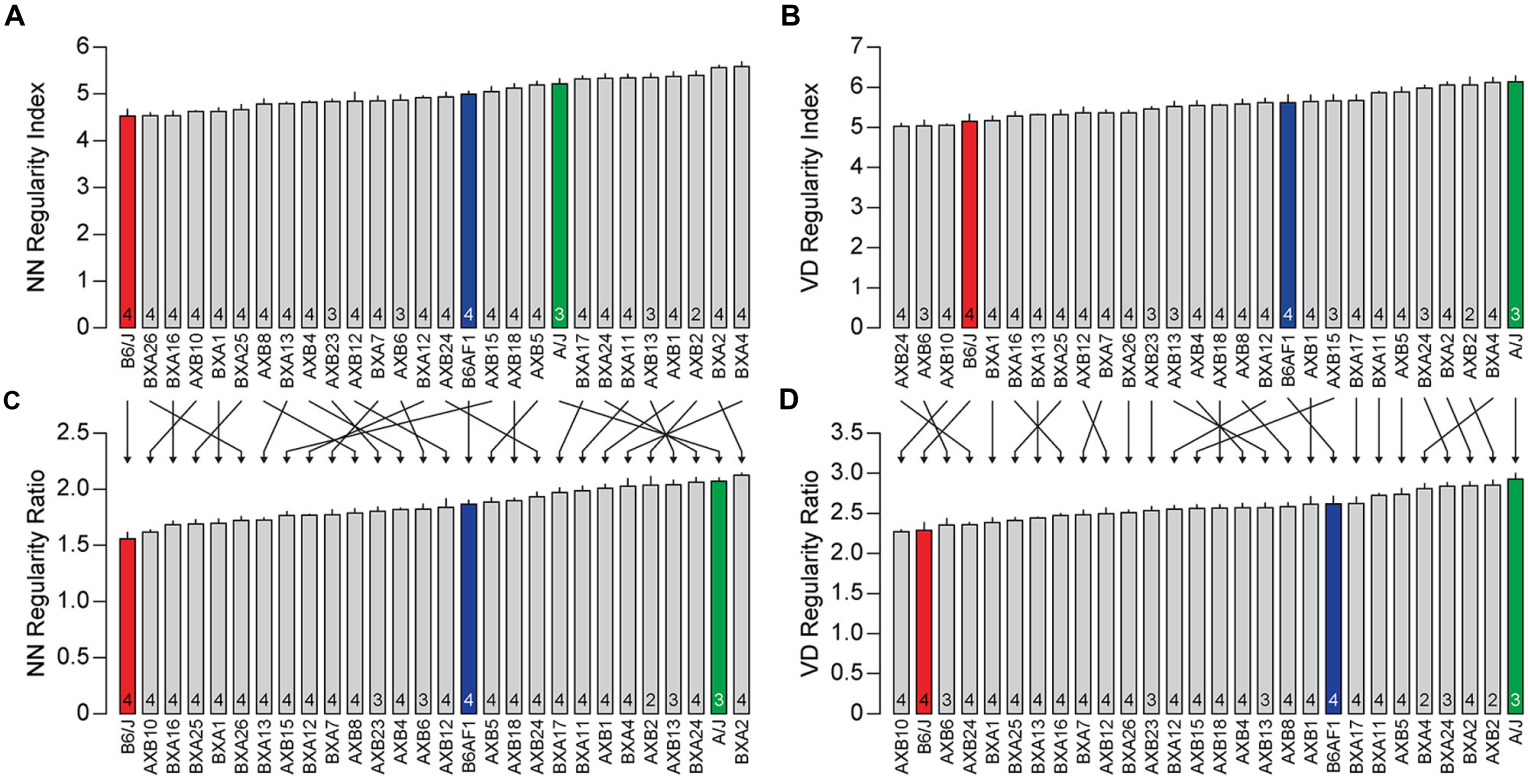
** (A,B)** The average NN and VD regularity index (±SE) for the three strains shown in **Figures 1 and 2** (colored bars) along with the 25 recombinant inbred strains of the AXB/BXA strain-set (gray bars). The n in each bar indicates the number of mice sampled. **(C,D)** The real/random regularity ratios for the same collection of strains. Note that the ordering of the strains has changed considerably due to this normalization, indicated by arrows, although more so for the NN analysis than the VD analysis.

To examine further the utility of this real/random regularity ratio, we have computed the regularity indexes for real and simulated random fields for each of the 25 recombinant inbred strains of mice of the AXB/BXA strain-set. The means and SE for each strain are summarized in **Table 1**. Their regularity indexes vary from 4.54 to 5.59 for the NN analysis and from 5.02 to 6.12 for the VD analysis (**Figures 3A,B**), with some strains having regularity indexes higher than the parental A/J strain or lower than the parental B6/J strain. As indicated above, however, these strains also vary in their average horizontal cell densities, and so the real magnitude of the differences in these regularity indexes, relative to random distributions of cells, is obscured. If those regularity indexes are normalized, as above, by computing the real/random regularity ratio (**Figures 3C,D**; **Table 1**), a conspicuous change is revealed in the strain distribution pattern, indicated by the arrows linking the bars in the histograms in **Figure 3**. This is most readily apparent by considering the relative positioning of the parental A/J strain in the NN analysis (green bar in **Figures 3A,C**), which has migrated to the higher extreme of this strain distribution pattern, there being one other strain with a higher real/random regularity ratio (the BXA2 strain). In short, the normalization has led to a re-ordering of the strains according to how regular they are relative to their density-matched random simulations.

**Table 1:** The averages and SE for the two regularity indexes, the two regularity ratios, and the packing factor for the two parental strains, the F1 strain, and the 25 recombinant inbred strains.

		NN Regularity Index		NN Regularity Ratio		VD Regularity Index		VD Regularity Ratio		Packing Factor	
Strain	*N*	Average	SE	Average	SE	Average	SE	Average	SE	Average	SE
C57BL/6J	4	4.53	0.14	1.56	0.06	5.15	0.17	2.29	0.10	0.316	0.005
A/J	3	5.22	0.07	2.07	0.03	6.14	0.14	2.93	0.07	0.352	0.003
B6AF1	4	5.00	0.07	1.87	0.03	5.62	0.19	2.62	0.09	0.339	0.004
AXB1	4	5.37	0.11	2.01	0.04	5.65	0.17	2.61	0.10	0.338	0.002
AXB2	2	5.40	0.10	2.04	0.08	6.06	0.21	2.85	0.06	0.360	0.000
AXB4	4	4.82	0.04	1.82	0.02	5.55	0.12	2.57	0.05	0.335	0.006
AXB5	4	5.19	0.09	1.89	0.04	5.88	0.14	2.74	0.08	0.340	0.003
AXB6	3	4.87	0.12	1.82	0.05	5.04	0.15	2.36	0.09	0.324	0.004
AXB8	4	4.79	0.11	1.79	0.04	5.59	0.11	2.58	0.06	0.324	0.005
AXB10	4	4.63	0.03	1.62	0.02	5.06	0.03	2.27	0.02	0.317	0.004
AXB12	4	4.85	0.20	1.84	0.08	5.36	0.15	2.50	0.07	0.338	0.004
AXB13	3	5.35	0.10	2.04	0.05	5.52	0.14	2.57	0.06	0.340	0.008
AXB15	4	5.05	0.12	1.77	0.04	5.66	0.15	2.56	0.05	0.337	0.003
AXB18	4	5.13	0.10	1.90	0.02	5.56	0.03	2.56	0.05	0.324	0.004
AXB23	3	4.84	0.06	1.80	0.04	5.46	0.06	2.54	0.05	0.330	0.005
AXB24	4	4.93	0.11	1.93	0.05	5.02	0.08	2.36	0.03	0.330	0.002
BXA1	4	4.63	0.09	1.70	0.04	5.17	0.13	2.39	0.06	0.323	0.005
BXA2	4	5.57	0.05	2.12	0.02	6.05	0.09	2.85	0.06	0.358	0.008
BXA4	4	5.59	0.11	2.03	0.07	6.12	0.14	2.80	0.07	0.315	0.006
BXA7	4	4.85	0.11	1.77	0.05	5.36	0.08	2.48	0.06	0.324	0.002
BXA11	4	5.35	0.08	1.98	0.05	5.87	0.04	2.73	0.03	0.321	0.004
BXA12	4	4.92	0.05	1.77	0.01	5.61	0.12	2.55	0.05	0.334	0.004
BXA13	4	4.79	0.04	1.72	0.02	5.31	0.03	2.44	0.02	0.321	0.003
BXA16	4	4.54	0.10	1.68	0.04	5.28	0.12	2.47	0.03	0.317	0.003
BXA17	4	5.32	0.07	1.97	0.04	5.67	0.16	2.62	0.08	0.334	0.003
BXA24	4	5.34	0.10	2.06	0.04	5.98	0.09	2.84	0.05	0.355	0.002
BXA25	4	4.66	0.12	1.69	0.04	5.31	0.13	2.41	0.04	0.326	0.005
BXA26	4	4.54	0.07	1.72	0.04	5.36	0.07	2.51	0.04	0.325	0.002

**FIGURE 4:**
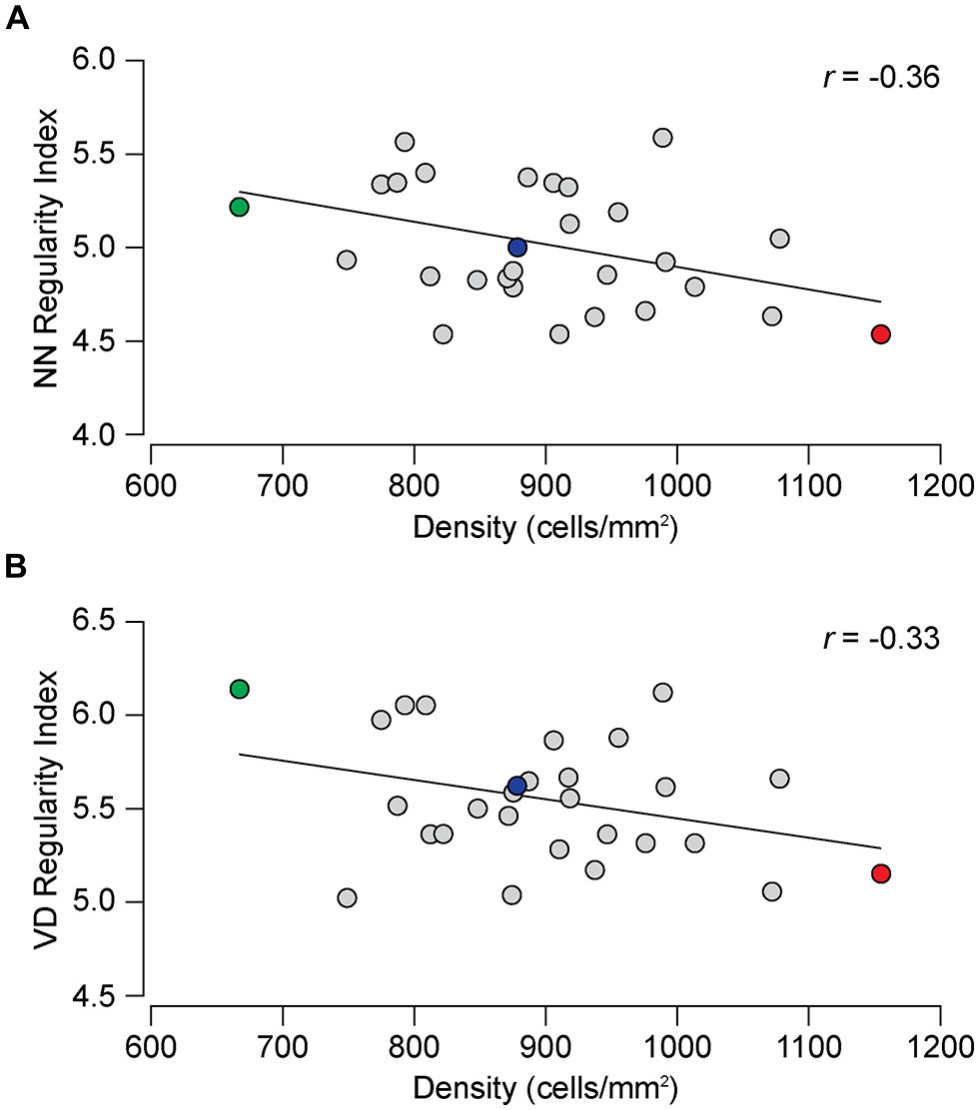
** (A)** The relationship between NN regularity index and average horizontal cell density across the 28 strains. The slight negative correlation is not significant, the Pearson correlation coefficient (*r*) being –0.36 (*p* = 0.06). **(B)** The correlation between the VD regularity index and horizontal cell density was not significant (*r*= –0.33, *p* = 0.08).

While **Figures 2A,B** might suggest that the difference in regularity is related to density, **Figures 4A,B** show no such relationship between the NN or VD regularity index and average horizontal cell density across this entire collection of strains. While there are slight negative relationships between the regularity indexes and density, they are non-significant (NN regularity index vs. density, *r* = –0.36, *p* = 0.06; VD regularity index vs. density, *r* = –0.33, *p* = 0.08). Many strains with identical average densities have conspicuously different regularity indexes, making clear that the differences in regularity index between the parental and F1 strains shown in **Figure 2** should be independent of their differences in density. Such variation in regularity should reflect differences in the effectiveness by which horizontal cells space themselves apart. Normalizing the regularity index yields a reordering of the strains (**Figures 3C,D**) that should better portend the actions of biological processes underlying this variation in the regularity of the horizontal cell mosaic.

**FIGURE 5:**
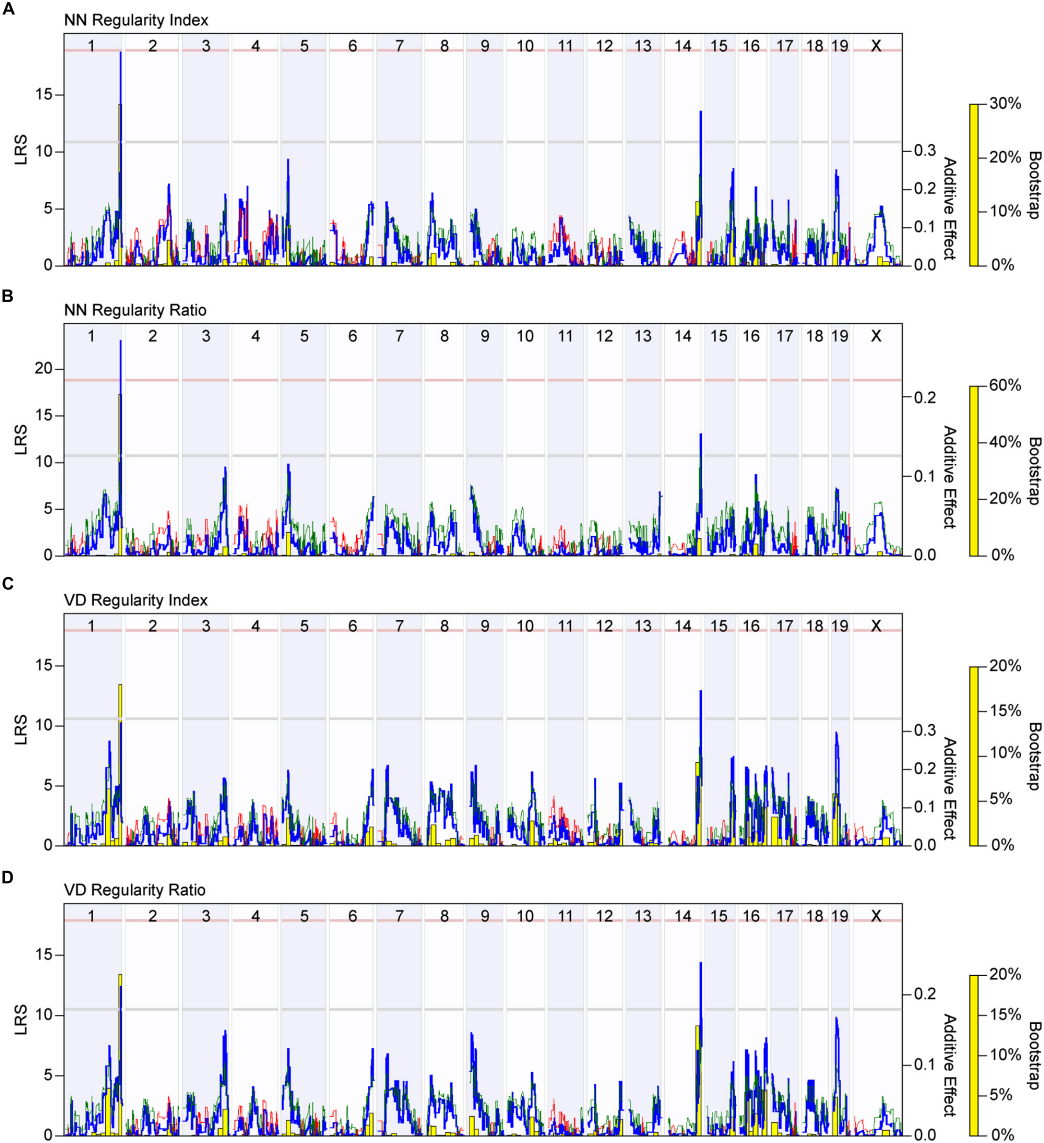
** (A)** Whole genome map for NN regularity index. The blue trace indicates the LRS across the mouse genome, indicating the strength of linkage between phenotype with genotype (left y-axis). The red and green traces indicate those locations across the genome where the presence of *B* vs. *A* alleles, respectively, correlate with an increase in trait values, the magnitude of which is indicated for each allele (additive effect; right y-axis). The gray and pink horizontal dashed lines indicate the suggestive (*p* < 0.63) and significant (*p* < 0.05) thresholds for LRS scores determined through permutation testing of the strain data. A significant QTL is detected on the distal end of Chr 1, while a suggestive QTL is detected on the distal end of Chr 14. The yellow bars indicate the bootstrap analysis (being the proportion of bootstrap samples mapping to a given locus), assessing the robustness of the mapping to any genomic locus. **(B)** Whole genome map for the NN real-random regularity ratio, with all conventions as in **(A)**. Note that the LRS score associated with the peak of the QTL on Chr 1 has increased, while the significant threshold has declined. **(C,D)** Whole genome maps (following the conventions as in **A**) for the VD regularity index and VD regularity ratio reveal the same two QTL on Chrs 1 and 14. Note that the LRS scores associated with each QTL increased after mapping the regularity ratio.

Further validation of this view is provided by mapping the variation in regularity across the 25 strains to the variation in haplotype composition across their genomes. **Figure 5A** shows the resultant whole genome map for the NN regularity index, indicating the presence of two QTL that each surpass the suggestive threshold defined by permutation testing (gray horizontal dashed line in **Figure 5A**), positioned at the distal ends of Chrs 1 and 14. At each of these loci, the presence of *A* alleles is associated with an increase in the NN regularity index. The QTL on Chr 1 is associated with an LRS score of 18.67, just below the significant threshold (pink horizontal dashed line in **Figure 5A**). The QTL on Chr 14 is associated with an LRS score of 13.51. **Figure 5B**, by contrast, shows the whole genome map produced using the NN real/random regularity ratio. Variation in this ratio trait also maps to the same pair of loci, but the locus on Chr 1 is now associated with an LRS score of 23.26 that far surpasses the significant threshold, the latter having declined slightly, both a consequence of the strain reordering. Minimal changes were observed with the linkage to the locus on Chr 14.

Variation in the VD regularity index across these strains also mapped to these same genomic loci on Chrs 1 and 14, with the presence of *A* alleles at each locus being associated with an increase in VD regularity index; the QTL on Chr 14, however, had an LRS score that was now higher than the QTL on Chr 1, the latter failing to reach the suggestive threshold (**Figure 5C**). Mapping the variation in the VD real/random regularity ratio increased the LRS scores for each of these QTL (from 13.04 to 14.41 for the peak on Chr 14; from 10.35 to 12.32 for the peak on Chr 1), elevating that on Chr 1 to surpass the suggestive threshold (**Figure 5D**). These results suggest that the real/random regularity ratio may be an effective means for comparing regularity across strains that exhibit a large variation in density.

**FIGURE 6:**
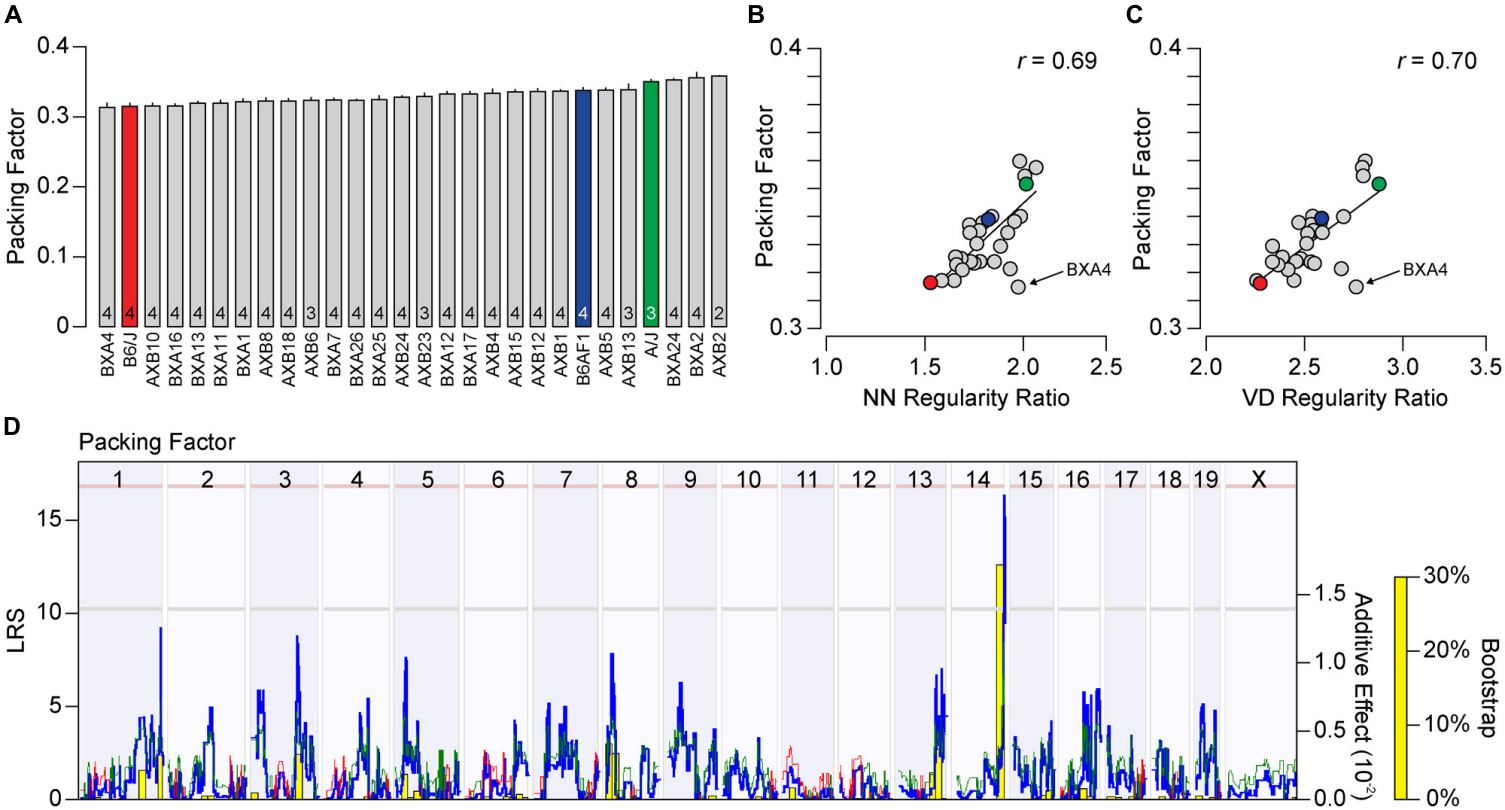
**(A)** Average packing factor (±SE) across the two parental strains, the F1 cross, and the 25 recombinant inbred strains. **(B,C)** The packing factor across the strains is significantly and positively correlated to both the NN and VD regularity ratio, with *r* values of 0.69 (*p* = 2.3 × 10^-5^) and 0.70 (*p* = 1.3 × 10^-5^), respectively. **(D)** Whole genome map, with conventions as in **Figure 5A**, for packing factor across the strains. A suggestive QTL on Chr 14 was observed, with a peak LRS nearing the significant threshold (pink horizontal dashed line). While the second highest LRS score was associated with the locus on Chr 1, it failed to cross the suggestive threshold (gray horizontal dashed line).

The packing factor is another measure of spatial patterning, describing how well a mosaic approximates a hexagonal lattice, which takes into consideration the density of each field. We therefore asked whether the variation in packing factor across the recombinant inbred strains would correlate to either of the regularity indexes or regularity ratios, and whether this variation would map to the same genomic loci or might identify entirely novel ones. The packing factor, like the regularity indexes, varied between the parental strains, with the A/J strain have a higher degree of packing than the B6/J strain (0.35 vs. 0.32), as well as across the recombinant inbred strains, ranging from 0.31 to 0.36 (**Figure 6A**). The packing factor across the strains was positively, and significantly, correlated to both regularity indexes and ratios, though to a greater extent for the regularity ratios (**Figure 6B**; *r* = 0.69, *p* = 2.3 × 10^-5^ for NN real/random regularity ratio; and **Figure 6C**; *r* = 0.70, *p* = 1.3 × 10^-5^ for VD real/random regularity ratio), although this association was not as great as the correlation between the two regularity ratios themselves (*r* = 0.83, *p* = 4.3 × 10^-9^). This variation in packing factor across the recombinant inbred strains mapped to the same genomic locus on Chr 14, associated with an LRS score of 16.45 approaching the significant threshold (**Figure 6D**). A hint of the locus on Chr 1 was also detected, although the LRS score at this locus did not pass the suggestive threshold. As before, the presence of *A* alleles at each of these loci was associated with an increase in the packing factor trait.

**FIGURE 7:**
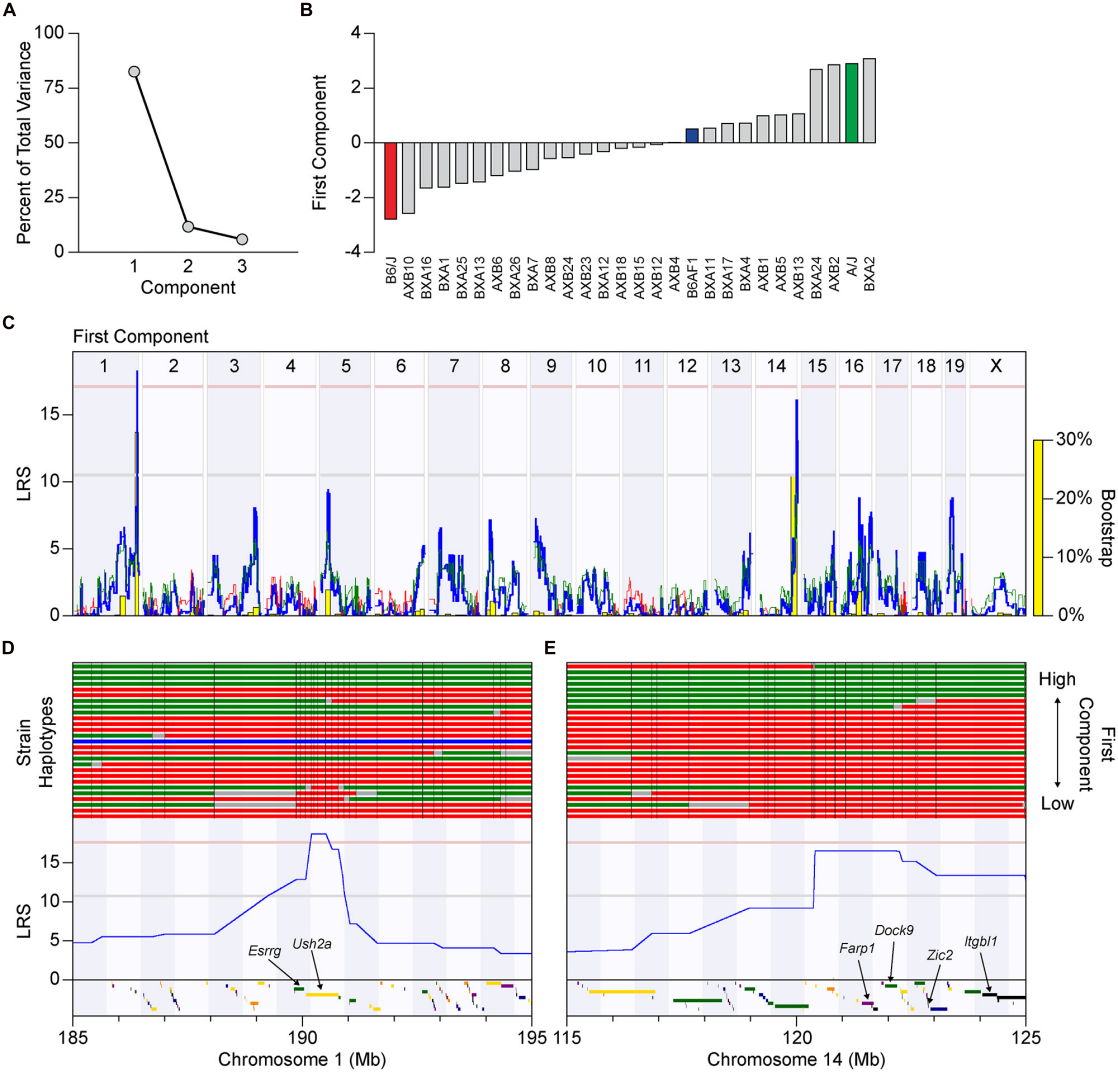
**(A)** Principal component analysis (PCA) using the NN regularity ratio, VD regularity ratio, and packing factor traits as variables, revealed a first principal component that accounted for over 80% of the total variance across these traits. **(B)** The value for each recombinant inbred strain along this first component was used to create a new trait that could be mapped. Notice that the two parental strains are located near the extremes of the distribution. **(C)** Whole genome map for the association between this first component with genotype across the strains, with conventions as in **Figure 5A**. Both previously identified QTL, on Chrs 1 and 14, have high LRS scores, with the former passing the significant threshold and the latter closely approaching it. **(D,E)** Haplotype maps of the 25 recombinant inbred strains and two parental strains through a 10 Mb region centered at the QTL on Chrs 1 and 14, with the *A* haplotype indicated in green, and the *B* haplotype in red. A single strain (AXB18) is heterozygous for *A* and *B* alleles at the Chr 1 locus, and is indicated in blue. The strains are ordered, from bottom to top, by their increasing value of the first component. Three genes (colored rectangles below each LRS trace) reside at the narrow genomic locus on Chr 1, while 25 genes are present at the locus on Chr 14, which were narrowed down to six top candidate genes based on bioinformatic analysis.

The difference in this map and those achieved by using the regularity ratios as traits might suggest that the spatial measures of regularity vs. packing are each modulated somewhat independently by distinctive biological processes. Yet given the high degree of correlation between these three traits (the NN and VD regularity ratios and the packing factor), we wondered whether the differences in these whole genome maps reflected the actions of a few unusual strains; the BXA4 strain, for example, had the lowest packing factor of any strain analyzed, yet had regularity ratios that were in the top quarter of all strains (**Figures 6B,C**). We consequently performed PCA to determine if a single component could account for most of the variance observed across the three traits. Indeed, the first principal component accounted for over 80% of the total variance (**Figure 7A**), with each of the three traits contributing equally. The distribution of recombinant inbred strains along the first principal component is shown in **Figure 7B**. Whole genome mapping of this new quantitative trait revealed the two previously elucidated QTL on Chr 1 and Chr 14, with LRS scores of 18.15 and 16.00, respectively, with the former QTL crossing the significant threshold and the latter falling slightly beneath (**Figure 7C**). We conclude that both loci contain genetic variants that contribute to the difference in horizontal cell patterning across the recombinant inbred and parental strains, but that there is no basis for concluding that the two loci modulate distinctive biological processes, for instance, one that modulates local spacing vs. another than coordinates patterning across larger distances.

It is of further interest to note that most of the variation observed across the recombinant inbred strains could be attributed to the magnitude of the effects at these two loci, regardless of which trait was analyzed. For instance, the additive effect of *A* alleles at the loci on Chrs 1 and 14 upon the NN regularity ratio was 0.25 and 0.19, respectively (e.g., **Figure 5B**), their summed effects equaling 79% of the range in this ratio across the strains (**Figure 3C**; **Table 2**). The same summed additive effects for the VD regularity ratio equaled 73%, while that for the packing factor equaled 71% (**Table 2**). Such a large proportion of the variation in all three traits being attributed to only two genomic loci may explain the somewhat step-like (rather than smooth) progression in trait values across the strains shown in **Figures 3C,D and 6A**, as well as that observed for the first principal component in **Figure 7B**.

**Table 2:** The range of variation in the two regularity ratios and the packing factor, and the magnitude of the two QTL effects for each trait, as well as their summed proportion of the range.

	Total variation across all strains^a^	Additive effect of two *A* alleles Chr 1 QTL^b^	Additive effect of two *A* alleles Chr 14 QTL^b^	Percent of total variation
NN Regularity Ratio	0.57	0.25	0.19	79%
VD Regularity Ratio	0.66	0.24	0.23	73%
Packing Factor	0.045	0.014	0.018	71%

*^a^From **Table 1**.*

*^b^From **Figures 5B,D*and*6D**.*

A list of all candidate protein-coding genes at these two loci is presented in **Table 3**. For each gene, a summary of protein structure and function was obtained from the Uniprot database^2^, while genetic variants present between the two parental strains were obtained from the Wellcome Trust Sanger Institute’s Mouse Genomes Project^3^. Top candidates had known functional roles in cell-to-cell communication, cytoskeletal rearrangement or transcriptional regulation, as well as genetic variants in regulatory regions (such as upstream, downstream, splicing, or untranslated regions), which may alter gene expression, and/or in protein-coding regions, which may affect protein sequence and function. The QTL on Chr 1 is extremely narrow, and of the genes at this locus, *Esrrg* and *Ush2a* were the most compelling (**Figure 7D**). *Esrrg* belongs to a family of constitutively active nuclear receptors that modulate transcription by binding to estrogen response elements, and is expressed in the nervous system, including the retina, throughout development (Hermans-Borgmeyer et al., 2000). *Ush2a* encodes the protein Usherin, mutations in which can lead to Usher syndrome, a developmental disease affecting both the visual and auditory pathways (Liu et al., 2007). Usherin is a single-pass transmembrane protein with a large extracellular domain that contains many fibronectin- and laminin-like domains, bearing similarity to Megf10 and Megf11, both of which have been shown to affect horizontal cell regularity (Kay et al., 2012). While *Ush2a* is thought to be expressed solely in photoreceptor cells in adult retinas, it is also expressed in prenatal development, potentially in developing horizontal cells. The QTL on Chr 14 encompasses a larger interval: top candidates include *Farp1, Dock9, Zic2,* and *Itgbl1* (**Figure 7E**). *Farp1* and *Dock9* both play a role in cytoskeletal dynamics, and could be potential regulators of horizontal cell movement, a suggested mechanism for achieving mosaic regularity. *Zic2* is expressed in the embryonic and early postnatal retina and has been shown to affect various processes, including retinal ganglion cell axon pathfinding and progenitor cell proliferation (Herrera et al., 2003; Watabe et al., 2011). Finally, *Itgbl1* encodes a secreted integrin-like protein that has several EGF-like repeat domains, which resemble those of the Megf proteins, although little is known about this protein aside from these structure similarities.

**Table 3:** Bioinformatic analysis of genes at the two identified QTL^**a**^, including known protein structure and function, as well as the presence of genetic variants between the A/J and B6/J parental strains.

Gene name	QTL	Transcriptional start site Mb (mm9)	Protein		Genetic variants (SNPs or INDELs)
			Class/type	Function	Regulatory	Protein-coding
*Esrrg*	Chr 1	189.821706	Nuclear receptor	Transcriptional activator	Yes	No
*Ush2a*	Chr 1	190.086717	Single transmembrane domain containing	Involved in hearing and vision	Yes	Yes
*Kctd3*	Chr 1	190.794977	Potassium channel tetramerization domain containing	Accessory subunit to HCN complexes	Yes	No
*Oxgr1*	Chr 14	120.418807	G-protein coupled receptor	Receptor for alpha-ketogluterate	Yes	Yes
*Mbnl2*	Chr 14	120.674891	RNA-binding	Regulates pre-mRNA splicing	Yes	No
*Rap2a*	Chr 14	120.877683	Small GTPase	Regulates cytoskeletal rearrangement	No	No
*4930424E08Rik*	Chr 14	121.036256	Unknown	Unknown	Unknown	Unknown
*Ipo5*	Chr 14	121.310416	Ran-binding	Nuclear import	No	No
*Farp1*	Chr 14	121.434796	Guanine nucleotide exchange factor	Regulates cytoskeletal rearrangement	Yes	Yes
*Stk24*	Chr 14	121.685563	Kinase	Promotes apoptosis	Yes	No
*Slc15a1*	Chr 14	121.858843	Twelve transmembrane domain containing	Oligopeptide transport	Yes	Yes
*Dock9*	Chr 14	121.941261	Guanine nucleotide exchange factor	Regulates cytoskeletal rearrangement	Yes	Yes
*Ubac2*	Chr 14	122.277828	Three transmembrane domain containing	Protein trafficking	Yes	No
*Gpr18*	Chr 14	122.310656	G-protein coupled receptor	Receptor for *N*-arachindonyl glycine	Yes	No
*Gpr183*	Chr 14	122.351553	G-protein coupled receptor	Receptor for oxysterol	Unknown	Unknown
*Timm8a2*	Chr 14	122.433896	Chaperone	Putative mitochondrial chaperone	Yes	No
*Tm9sf2*	Chr 14	122.506304	Nine transmembrane domain containing	Putative small molecule transporter	Yes	No
*Clybl*	Chr 14	122.580916	Enzyme	Cellular metabolism	Yes	Yes
*2810433H14Rik*	Chr 14	122.777219	Unknown	Unknown	Unknown	Unknown
*Zic5*	Chr 14	122.858382	Zinc finger transcription factor	Nervous system development	No	No
*Zic2*	Chr 14	122.874606	Zinc finger transcription factor	Nervous system development	Yes	No
*Pcca*	Chr 14	122.933550	Enzyme	Cellular metabolism	Yes	No
*Ggact*	Chr 14	123.290082	Enzyme	Degrades protein cross-links	Unknown	Unknown
*Tmtc4*	Chr 14	123.318197	Twelve transmembrane domain containing	Unknown	Yes	No
*Nalcn*	Chr 14	123.675863	Twenty four transmembrane domain containing	Non-selective cation leak channel	Yes	Yes
*Itgbl1*	Chr 14	124.059362	Secreted Integrin-like	Unknown	Yes	Yes
*Fgf14*	Chr 14	124.377513	Intracellular fibroblast growth factor	Nervous system development	Yes	No
*4930404O17Rik*	Chr 14	125.129541	Unknown	Unknown	Unknown	Unknown

*^a^Gene list was derived from the intervals 189.866756–190.912793 Mb on Chromosome 1 and 120.405786–124.975961 Mb on Chromosome 14.*

## DISCUSSION

The non-random distribution of like-type neurons within a structure has been considered a defining feature of neuronal populations (Cook, 2003), yet the molecular mechanisms that establish these “mosaics” are relatively unknown. Spatial statistics such as the regularity index and the packing factor can be used to describe the orderliness of these distributions. By treating these statistics as quantifiable traits, one can map such variation in spatial patterning across mouse strains to the variation in haplotype structure of their genomes. This, in turn, facilitates the pursuit of candidate genes and their variants that regulate cell patterning. The present study has adopted this approach, revealing two distinct genomic loci on Chrs 1 and 14 that control the patterning of the horizontal cell mosaic.

The regularity index, being either the mean NN distance or VD area divided by the SD of those values, has a lower bound defined by a theoretical random distribution of dimensionless points, but has no upper bound, as the variance in either measure approaches zero. While it effectively describes the spatial order of a two-dimensional point pattern, it has generally been used to indicate simply that a mosaic is more regular than a random point pattern. Beyond this, it has seen little comparative application beyond demonstrations that the regularity index is altered by experimental or genetic manipulations. Interpreting such alterations, of course, requires a consideration of whether the manipulation also changes the number of elements in the mosaic, a common variable following such perturbations, a variable also observed across different strains of mice (Keeley et al., 2014a).

In the present study, we consider directly the role that density plays upon constraining a random simulation of horizontal cells, and how computing the regularity index without taking this into consideration underestimates the differences in regularity between mosaics. Indeed, we go on to show that, by calculating the real-to-random regularity ratio, we map more robust QTL with stronger linkage on Chrs 1 and 14. These results would suggest that the real/random regularity ratio, be it derived from the NN analysis or the VD analysis, more acutely discriminates strains by the actions of biological processes that space cells apart.

### THE REAL/RANDOM REGULARITY RATIO

This transformation of the data, creating the real/random regularity ratio, is not without its caveats and limitations. For instance, this ratio will by necessity change as a function of development: before the retina has achieved its adult size, but after the cells have approached their mature diameters, so the regularity index of random simulations for these denser mosaics in smaller (younger) retinas will be larger, yielding lower real/random regularity ratios relative to more mature retinas, even when the patterning of the real distributions does not differ (e.g., see Raven and Reese, 2002, for a comparison of real distributions vs. random simulations of the horizontal cell mosaic in B6/J at 3 weeks of age). Likewise, some experimental or genetic manipulations that alter the patterning of a retinal mosaic also affect somal size substantially (Cantrup et al., 2012). In such instances, particularly where cell density and retinal area change as well, the calculation of the real/random regularity ratio would likely provide little additional insight into understanding the factors controlling nerve cell spacing.

In the present study, we have compared the patterning of horizontal cells across mature mouse retinas that show little variation in retinal area but conspicuous twofold variation in cell number, consequently yielding large differences in horizontal cell density (the slight differences in retinal area across the strains, like the slight differences in age, show no significant correlation with density, regularity index, regularity ratio nor packing factor). While horizontal cells are notoriously plastic (Poché and Reese, 2009), and can hypertrophy to an excess of twice their normal somal area, for example, in the absence of *Pten* (Cantrup et al., 2012), they do not exhibit any change in soma size across the present strains (**Figure 1**). We have, consequently, carried out this transformation of the regularity index where only cell density varies across the strains. That this transformation might be meaningful for understanding the biology of mosaic order is suggested by the reordering of the strains, yielding stronger linkage between phenotype with genotype (**Figure 5**). While it is true that the QTL on Chrs 1 and 14 were detected without transforming the data in this manner, in one of the cases (the QTL on Chr 1 for VD regularity index), it was sub-threshold and likely would have gone unexamined in the absence of the other analyses. The present results, therefore, would substantiate the principle that where density varies conspicuously in the absence of other differences in the population, correcting for this effect of density upon spacing should enhance detection of genomic linkage.

Across the recombinant inbred strains, computing the regularity ratio had a greater effect on reordering the strains in the NN analysis than the VD analysis (**Figure 3**), since random simulations for the latter measure varied less across fields with different densities (**Figure 2**). This fact, due to the relatively greater constraining effect of soma size upon the linear NN measure than upon the areal VD measure, along with the high correlation between the two measures of regularity, might lead one to believe that the VD regularity index is a sufficient and complete measure of mosaic patterning. However, despite the more modest changes in strain order between the VD regularity index and regularity ratio, the ratio statistic still increased the strength of linkage at each QTL. And while both regularity index were correlated, the QTL on Chromosome 1 was most prominent using the NN spatial statistic. These results suggest that both the NN and VD regularity ratios provide valuable information about horizontal cell regularity, and should be used in a complementary fashion.

### THE PACKING FACTOR

We also compared the results obtained using the real/random regularity ratio with those from the packing factor analysis (Rodieck, 1991), which also varied across the strain-set. One attraction of the packing factor is that it has both a lower and an upper bound, ranging from 0 (a random distribution of dimensionless points) to 1 (being a perfect hexagonal matrix). Another is that this measure is normalized for density, as it is the ratio of the effective radius to the maximal radius permissible for a hexagonal lattice of identical density (Rodieck, 1991). We found that the variation in packing factor across the strains showed the strongest linkage on Chr 14, whereas the QTL on Chr 1 failed to cross the suggestive threshold. The packing factor, therefore, did not completely recapitulate the same genome maps that were generated using the real/random regularity ratios, particularly that for the NN analysis. It is important to bear in mind that the packing factor is not a measure of regularity (Rodieck, 1991); rather, it is a distinctive measure of patterning, and therefore might be indicative of distinctive biological processes that contribute to nerve cell patterning. Because the horizontal cell mosaic in the mouse retina does not evidence the presence of higher order patterning as found in a lattice, even with substantial jitter (Reese and Keeley, 2014), and since the packing factor was so strongly correlated with either regularity ratio, the present results would suggest that the measures of regularity and packing are assessing similar qualities present in these real mosaics. The PCA would support this conclusion. By using this PCA to reduce the dimensionality of the data, a single trait emerges, capturing most of the variation in patterning across the recombinant inbred and parental strains. Mapping the variation in this principal component, we recapitulated both genomic loci, each showing strong linkage (evidenced through permutation testing) and reproducibility (evidenced by bootstrap testing). Each locus must contain genetic variants that affect the patterning of horizontal cells, presumably by modulating their intercellular spacing at the local level.

### GENETIC VARIANTS MODULATE HORIZONTAL CELL PATTERNING INDEPENDENT OF DENSITY

The regularity of the horizontal cell mosaic is not significantly correlated with the variation in horizontal cell density, and the mapping of the variation in horizontal cell patterning does not coincide with the genomic locus on Chr 13 mapped by the variation in horizontal cell number or density (Whitney et al., 2011). Horizontal cells are known to interact with one another as they assemble their mosaics and initiate their differentiation (Raven et al., 2005b; Poché et al., 2008; Huckfeldt et al., 2009), and they use Megf10 and Megf11 to drive that assembly into regular distributions (Kay et al., 2012). The present QTL mapping exercise indicates that genetic variants on Chrs 1 and 14 should modulate this process. Of the candidate genes at these loci, the gene at the very peak of the QTL on Chr 1, *Ush2a*, is notable because of the structural similarities of the Usherin protein to the Megf proteins. While it is well-known for its structural role in the connecting cilium of developing photoreceptors, it is expressed in developing retina at very early stages (Huang et al., 2002), during the period when horizontal cells are being generated, and well before outer segment formation. Whether it is expressed transiently in developing horizontal cells remains to be seen. Additionally, the guanine nucleotide exchange factor Farp1 is a particularly intriguing candidate at the peak of the QTL on Chr 14 due to its role in dendritic morphogenesis (Cheadle and Biederer, 2012), as well as its interaction with Sema6A and PlexA4 proteins (Zhuang et al., 2009), both shown to be involved in horizontal cell development (Matsuoka et al., 2012). Future studies will examine the candidates at these two genomic loci in further detail to understand the genetic and molecular control of horizontal cell patterning in the developing retina.

## AUTHOR CONTRIBUTIONS

Patrick W. Keeley and Benjamin E. Reese designed research; Patrick W. Keeley performed research. Patrick W. Keeley and Benjamin E. Reese analyzed data; Patrick W. Keeley and Benjamin E. Reese wrote the paper.

## Conflict of Interest Statement

The authors declare that the research was conducted in the absence of any commercial or financial relationships that could be construed as a potential conflict of interest.

## Footnotes

1 http://www.genenetwork.org

2 http://www.uniprot.org/

3 http://www.sanger.ac.uk/resources/mouse/genomes/
